# Generality of a Paired-Stimulus Preference Assessment for Identifying Reinforcing Forms of Social Interaction

**DOI:** 10.3390/bs16040543

**Published:** 2026-04-06

**Authors:** Zoë A. D. Newman, Eileen M. Roscoe, Ali C. Schaefer

**Affiliations:** 1 Regis College Autism Center, Weston, MA 02493, USA; 2 Department of Psychology, Western New England University, Springfield, MA 01119, USA; eroscoe@necc.org; 3 The New England Center for Children, Southborough, MA 01772, USA; 4 Centria Autism, Farmington Hills, MI 48334, USA; alicschaefer@gmail.com

**Keywords:** social interaction, preference assessment, skill acquisition, reinforcement

## Abstract

Although social interaction can function as an effective reinforcer during skill acquisition for individuals with autism spectrum disorder (ASD), preferred forms may vary across individuals and tasks. This study evaluated the extent to which a paired-stimulus preference assessment (PSPA) predicted the reinforcing efficacy of different forms of social interaction across tasks of increasing complexity. Two individuals with ASD participated. A pictorial PSPA identified highly preferred (HP) and less preferred (LP) social interactions. Three reinforcer assessments evaluated the relative and absolute reinforcing effects of HP and LP social interactions for (a) a simple arbitrary response, (b) a complex arbitrary response chain, and (c) a complex socially relevant response chain. Across assessments, HP social interactions produced more efficient acquisition than LP social interactions and prompting only. LP social interactions functioned as reinforcers when HP alternatives were unavailable. These findings suggest PSPA outcomes may predict differences in relative reinforcer efficacy while highlighting the importance of evaluating both relative and absolute reinforcing efficacy.

## 1. Introduction

Social interaction can be an effective reinforcer during skill acquisition programs for individuals with autism spectrum disorder (ASD; Kang et al., 2013; Morris & Vollmer, 2020c). Various forms of social interaction have been shown to reinforce a range of responses, including mands (Athens & Vollmer, 2010), simple motor responses (Kelly et al., 2014), and prosocial behavior (e.g., praise statements; Strohmeier et al., 2018). Social interaction offers several practical advantages as a reinforcer: it is naturally embedded within educational and community environments, readily available, and can often be delivered rapidly without disrupting instruction. Social interaction combined with leisure activities can be more preferred relative to solitary leisure activities (Kanaman et al., 2022). Because social interaction is commonly present across settings (e.g., classrooms, job sites, and homes), its use as a reinforcer may facilitate generalization and maintenance of learned skills.

Despite these advantages, social interaction is not a unitary stimulus class. Topographies of social interaction (e.g., physical contact, vocal exchanges, shared activities) may vary substantially in reinforcing value across individuals. Moreover, the preference for different forms of social interaction may be influenced by variables such as the person providing the social interaction (R. Huntington & Schwartz, 2022). Preferred forms of social interaction are often idiosyncratic, underscoring the importance of systematic methods for identifying which interactions are most likely to function as effective reinforcers for a given individual. Several preference assessment procedures have been adapted for evaluating social interaction, including multiple-stimulus without replacement assessments (MSWO; Nuernberger et al., 2012), modified response restriction procedures such as the Social Interaction Preference Assessment (SIPA; Morris & Vollmer, 2019), and paired-stimulus preference assessments (PSPAs; Kelly et al., 2014). Because social interactions are actions performed by the experimenter rather than tangible stimuli, they can be difficult to present concurrently. Therefore, preference assessments that require the simultaneous presentation of no more than two stimuli (e.g., PSPAs) may be particularly practical. In addition, representative stimuli—such as photographs (Goldberg et al., 2023; Kelly et al., 2014), videos (Curiel & Poling, 2019; R. N. Huntington & Higbee, 2018; Davis et al., 2022), graphic-interchange-format images (GIFs, Morris & Vollmer, 2020b), or icons (Morris & Vollmer, 2019, 2020a)—can be used to depict social interactions, allowing for systematic comparison of alternatives that cannot be physically presented simultaneously.

Picture-based preference assessments for social interaction have been frequently used in previous research (Morris et al., 2023) and have been useful for identifying social interactions that are reinforcers (e.g., Davis et al., 2022; Goldberg et al., 2023; Harper et al., 2021; Kelly et al., 2014; Lang et al., 2014; Morris & Vollmer, 2020b). Kelly et al. (2014) provided a noteworthy demonstration of a pictorially based PSPA for identifying preferred forms of social interaction. In that study, indirect and direct pre-assessments were used to identify candidate social interactions, which were then depicted using photographs of the Experimenter delivering each interaction. These photographs were included in a PSPA along with a no-interaction control. The assessment produced clear and stable preference hierarchies of social interaction. Subsequent reinforcer assessments demonstrated that highly preferred social interactions were relatively more reinforcing than less preferred social interactions and the control condition. However, although less preferred social interactions often functioned as reinforcers when evaluated independently, preference hierarchies did not consistently predict reinforcing efficacy relative to the control.

One potential explanation for this outcome is that the target behavior used by Kelly et al. (2014) required minimal response effort. The dependent variable (a mand) may have been sufficiently low in response effort that less preferred social interactions maintained responding when higher-preference alternatives were unavailable. Morris et al. (2023) found that previous research that included reinforcer assessments of social interaction does not frequently include skill acquisition tasks. As a result, the extent to which preference assessment outcomes predict reinforcing efficacy for more complex skills remains unclear. This limitation is important because much of skill acquisition programming involves teaching response chains that include multiple steps and varying response topographies. Understanding whether preference hierarchies for social interaction predict reinforcing efficacy for complex tasks is therefore of conceptual and applied significance.

The efficiency of skill acquisition may be affected by different components of teaching procedures, including the quality of the reinforcer. For example, Lang et al. (2014) compared the percentage of correct responses during discrete trial training with highly preferred and less preferred social interactions. For the two participants, the highly preferred social interaction was associated with more consistent correct responses and lower levels of challenging behavior relative to the less preferred social interaction. Similarly, Morris and Vollmer (2020c) evaluated social interactions as consequences during six-step response chains (i.e., building block structures). The results showed that all three participants reached mastery criteria in fewer sessions when the highly preferred social interaction was delivered as the consequence compared to the less preferred social interaction. Together, these studies showed that preference assessment outcomes can predict the reinforcing efficacy of social interaction when teaching more effortful skills. However, the multi-step response chains in Morris and Vollmer were arbitrary tasks, and the generality of social interaction as a consequence for teaching more complex socially relevant skills remains unknown.

Seaver and Bourret (2014) evaluated the generality of effective teaching intervention components with a complex, arbitrary task and clinically relevant tasks. Seaver and Bourret compared three prompt types (model, vocal plus gestural, and physical) and three prompt-fading procedures for increasing a complex arbitrary task (i.e., completing an eight-step LEGO structure). The authors then compared the most and least effective combinations of prompt type and prompt-fading procedure for two clinically relevant tasks (e.g., making trail mix and setting the table). The most effective combination identified with the LEGO structures was also the most effective for the clinically relevant tasks. These procedures with concurrent teaching of response chains may be useful when comparing different social interactions as a reinforcer for teaching complex and socially relevant skills.

The purpose of the current study was to replicate and extend previous research in several ways. First, the experimenters replicated the pre-assessments and PSPA described by Kelly et al. (2014) to identify a highly preferred (HP) and a less preferred (LP) form of social interaction. Second, the experimenters extended Kelly et al. (2014) by assessing the generality of the PSPA for identifying social interactions to use as consequences for complex and socially relevant skills. Third, the experimenters replicated and extended Seaver and Bourret (2014) by using a similar skill acquisition comparison procedure to compare the relative reinforcing efficacy of different forms of social interaction.

## 2. Method

### 2.1. Participants and Setting

Two individuals diagnosed with autism spectrum disorder (ASD), who attended a residential school, participated. Participants were recruited based on caregiver reports indicating a preference for multiple forms of social interaction. Marley (pseudonym) was a 17-year-old boy who communicated vocally in one- to three-word phrases and full sentences. Alex (pseudonym) was a 14-year-old boy who communicated vocally using full sentences.

Prior to participation, both individuals demonstrated mastery of prerequisite conditional discrimination skills during an auditory-to-visual matching-to-sample task (i.e., identifying actions as a listener, Morris & Vollmer, 2020a). During this task, three photographs depicting the experimenter engaging in activities (e.g., reading a book, talking on the phone, walking on a sidewalk) were presented, and the participant was asked to touch the card showing the spoken action (e.g., “Which picture shows a woman reading?”). Both participants met the mastery criterion of at least 90% correct responding across two consecutive sessions.

Sessions were conducted in a research room (4.6 m × 4.6 m) located at the school, in the participants’ classroom at the school, or in the participants’ bedroom at the residential facility. Sessions were conducted two to four days per week and lasted approximately 1–2 h.

### 2.2. Pre-Assessments

Three pre-assessments, similar to those described by Kelly et al. (2014), were conducted to identify the topographies of social interaction to include in the preference assessment array. These assessments were conducted sequentially and included an indirect assessment, a descriptive assessment, and a motivating operation manipulation assessment. We identified two social interactions from the results of each pre-assessment to include in the preference assessment array. If a social interaction was identified across multiple pre-assessments, that social interaction was included in the array in addition to the two unique social interactions from each pre-assessment. Table 1 lists descriptions of the social interaction topographies included in the preference assessment array for each participant and the pre-assessment that informed their selection.

#### 2.2.1. Indirect Assessment

Two caregivers who worked with each participant at least four days per week for a minimum of three months served as informants. Each informant was interviewed individually using the Social Stimuli Questionnaire developed by Kelly et al. (2014). The experimenter asked both closed- and open-ended questions to identify preferred topographies of social interaction and to develop operational definitions of each topography.

During the closed-ended portion of the questionnaire, a variety of forms of social interaction (e.g., hand holding, physical redirection, clapping, statements of concern) were listed. For each form, informants were asked to report how frequently it occurred using a three-point scale: never (1), sometimes (2), or always (3). For social interactions reported to occur sometimes or always, informants were asked to indicate whether the interaction was preferred by the participant (“yes” or “no”).

During the open-ended portion of the questionnaire, informants were asked to list additional forms of social interaction not included in the closed-ended portion. For each interaction listed, informants again reported frequency of occurrence and whether the interaction was preferred using the same criteria described above. For both participants, at least one form of social interaction that met inclusion criteria for the preference assessment array was identified through this open-ended portion (e.g., head rubs for Marley and “copy me” for Alex).

For social interactions rated as always occurring and preferred, informants were asked to rank order the interactions from most preferred to least preferred. The highest-ranked social interactions were selected for inclusion in the preference assessment array (see Table 1). When rankings differed across informants, the social interaction with the highest median rank was selected (e.g., a social interaction ranked 3 and 3 across informants was selected over a social interaction ranked 1 and 7 across informants). Based on these rankings, arm tickles and head rubs were identified for Marley; tickles and high-fives were identified for Alex. A third social interaction (“copy me”) indicated by the indirect assessment was included in Alex’s preference assessment because it was also identified in the descriptive assessment (described below).

#### 2.2.2. Descriptive Assessment

Three 15 min video samples were collected, depicting each participant interacting with caregivers across various activities in their typical environment. Prior to formal data collection, the experimenter reviewed the video samples and recorded all topographies of social interaction delivered by the caregiver as a consequence (i.e., contingent on participant responding). During this review, the experimenter also noted variations in each topography and developed clear operational definitions for use in subsequent data collection.

These operational definitions were provided to primary and secondary observers, who independently recorded the frequency of each social interaction in 10 s bins during each of the three 15 min sessions. Data were summarized as the mean frequency of each social interaction across sessions. Mean count-per-interval interobserver agreement (IOA) was assessed for 100% of sessions. Mean IOA was 96.3% (range, 90.3–100%) for Marley and 98.9% (range, 98.3–100%) for Alex.

Based on these data, the two social interactions with the highest average frequency of occurrence were selected for inclusion in the preference assessment array (see Table 1). For Marley, these interactions were praise and eye contact. For Alex, these interactions were silly sounds and praise.

#### 2.2.3. Manipulation of the Motivating Operation

This assessment consisted of two 5 min sessions. During each session, the experimenter turned away from the participant, appeared occupied, and did not initiate interaction. If the participant emitted a mand for social interaction, the experimenter delivered the requested form of social interaction for 3–5 s. For example, if the participant requested to converse with the experimenter, the experimenter responded by making statements, asking questions, commenting on participant statements, or answering participant questions for 3–5 s. No programmed consequences were delivered for unprompted participant behavior other than mands for social interaction.

Observers recorded the frequency of participant-emitted vocal (e.g., “head rubs on my forehead, please”) and nonvocal (e.g., pulling the experimenter’s hand) mands for social interaction. Total count IOA was assessed for 100% of sessions. Mean IOA was 97.6% (range 95.2–100%) for Marley and 93.8% (range 92.7–94.9%) for Alex.

The two forms of social interaction requested most frequently during the motivating operation manipulation were included in each participant’s preference-assessment arrays (see Table 1). For Marley, these interactions were preferred non-work topics and schedule-related topics. For Alex, these interactions were eye contact and schedule-related comments.

## 3. Preference Assessments

### 3.1. Procedures

A series of PSPAs (Kelly et al., 2014) were conducted to identify a highly preferred (HP) and a less preferred (LP) form of social interaction for each participant. The assessment array included six or seven social interactions and a no-interaction control stimulus. Photo cards (12.7 × 17.8 cm) were used as representative stimuli for each social interaction. Each card depicted the experimenter delivering a specific form of social interaction to the participant (e.g., high-five) in the same setting in which PSPA sessions were conducted. A blank white card (12.7 × 17.8 cm) served as the control stimulus. For social interactions that primarily involved vocal exchanges, the photographs included an overlaid dialog box specifying the type of vocal interaction (e.g., “copy me,” “first then”) to increase the discriminability of the different social interactions (e.g., Lang et al., 2014).

Prior to the first PSPA session conducted each day, the experimenter conducted exposure trials to ensure that participants contacted the contingencies associated with each card. During exposure trials, the experimenter presented each card individually, prompted the participant to select the card, and delivered the corresponding social interaction or no interaction (control). One PSPA was conducted per session, and sessions were conducted 1–2 times per week.

During PSPA sessions, cards were presented in pairs such that each card was paired once with every other card in the array in a quasi-random order. At the start of each trial, the experimenter presented two cards and instructed the participant to “pick one.” Selection was defined as touching a card with any part of the hand. Following a selection, the unselected card was removed, and the experimenter delivered the consequence associated with the selected card (i.e., the corresponding social interaction or no interaction) for 3–5 s.

The HP social interaction was defined as the interaction associated with the highest percentage of selections across trials, and the LP social interaction was defined as the interaction associated with the lowest percentage of selections. At least three complete PSPA series were conducted for each participant. If consistent identification of the HP and LP social interactions was observed across three consecutive series, PSPA outcomes were considered stable. In other words, stability criteria were met if the same social interaction was selected on more than 80% of trials (i.e., HP social interaction) and the same social interaction was selected on the fewest trials (i.e., LP social interaction) for three consecutive PSPA series. For one participant, Alex, a fourth series was conducted to achieve stability across three consecutive series.

Observers recorded card selections during each trial. Data were summarized as the percentage of trials in which each card was selected. Trial-by-trial IOA was assessed for 50% of trials for each participant, and mean agreement was 100% for both Marley and Alex.

### 3.2. Results and Discussion

**Figure 1:**
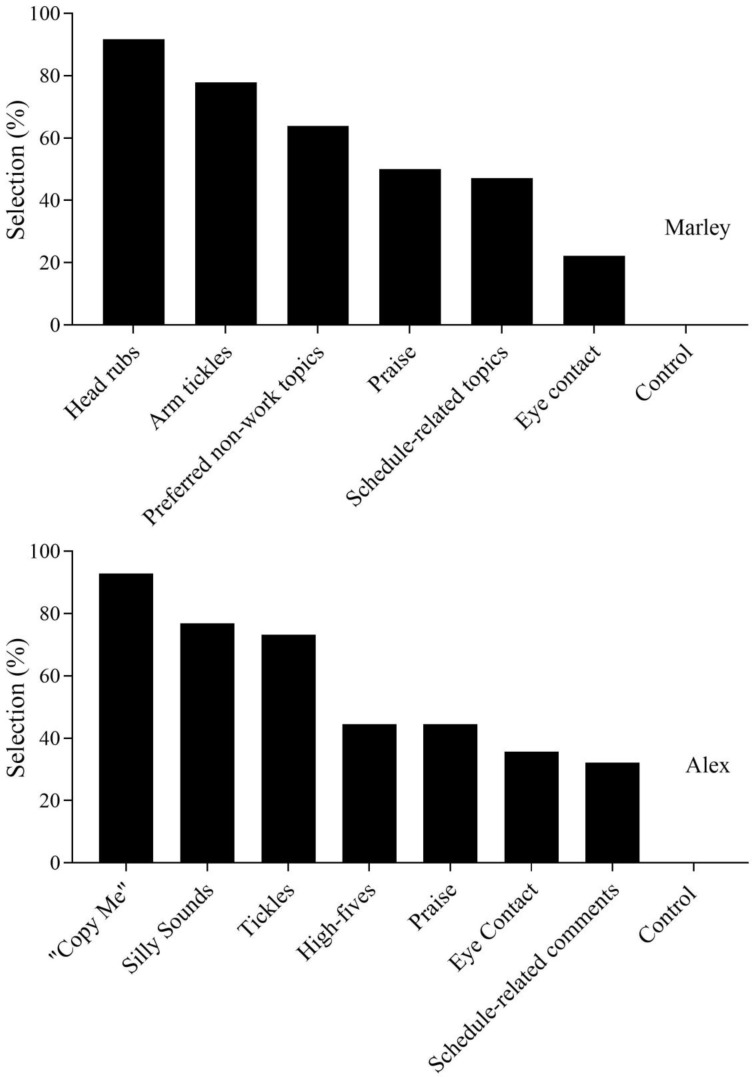
The results of the paired-stimulus preference assessment for each participant.

Figure 1 depicts PSPA outcomes for both participants. Marley (top panel) most frequently selected head rubs (*M* = 91.7%) and least frequently selected eye contact (*M* = 22.2%) and the control stimulus (*M* = 0%). Alex (bottom panel) most frequently selected “copy me” (*M* = 92.9%) and least frequently selected schedule-related comments (*M* = 32.2%) and the control stimulus (*M* = 0%).

Three reinforcer assessments were conducted to evaluate the generality of the PSPA outcomes across target skills of increasing complexity and social relevance. Across all reinforcer assessments, three consequences were compared: a highly preferred social interaction, a less preferred social interaction, and a no-interaction control.

Reinforcer Assessment 1 replicated Kelly et al. (2014) by evaluating the relative and absolute reinforcing effects of HP and LP social interactions for a simple target response. Reinforcer Assessment 2 extended Kelly et al. (2014) and Seaver and Bourret (2014) by evaluating the reinforcing efficacy of HP and LP social interactions for complex arbitrary response chains. Reinforcer Assessment 3 further evaluated the generality of PSPA outcomes by examining the use of HP and LP social interactions to teach complex socially relevant response chains.

## 4. Reinforcer Assessment 1: Simple Arbitrary Task

This assessment examined the reinforcing efficacy of highly preferred (HP) and less preferred (LP) social interactions for a simple, arbitrary response (i.e., target touching).

Target touching was defined as moving any part of the hand from the target on one side of the board to the target on the opposing side without interruption. Observers recorded the frequency of target touching during 5 min sessions using 10 s bins. Data were summarized as the rate of responding (responses per minute; rpm) for each session. Sessions were conducted 1–2 times per week, and 5–10 sessions were conducted per day.

Mean count-per-interval interobserver agreement (IOA) was assessed for 30% of sessions for Marley and 36.7% of sessions for Alex. IOA was calculated by dividing the 5 min session into 10 s intervals and comparing the frequency recorded within each interval across two independent observers. For each 10 s interval, the smaller frequency was divided by the larger frequency, and the quotients for all intervals were summed and then multiplied by 100. Mean IOA across all sessions was 99.6% for Marley (range, 98.3–100%) and 98.4% for Alex (range, 93–100%).

A reversal design with concurrent schedules was used, including a three-response-option (HP, LP, control) and two-response-option (LP, control) baseline and reinforcement phases. During baseline phases, no programmed consequences were delivered. During reinforcement phases, responding on the HP and LP targets produced the corresponding social interaction on an FR 1 schedule, and responding on the control target produced no interaction.

The three-response-option phases allowed for evaluation of the relative reinforcing effects of HP and LP social interactions compared to a no-interaction control. The two-response-option phases permitted evaluation of the absolute reinforcing efficacy of the LP social interaction when the HP alternative was unavailable. During the three-response-option baseline (A) and reinforcement (B) phases, three response options associated with HP, LP, and control consequences were concurrently available. During the two-response-option baseline (C) and reinforcement (D) phases, only the LP and control response options were available. For Marley, the sequence of phases was ABADBCD. For Alex, the sequence of phases was ABABCDCD.

Prior to the first baseline session, the experimenter conducted exposure trials to ensure that participants contacted the contingencies associated with each response option. During exposure trials, the experimenter modeled the target-touching response and provided the opportunity for the participant to respond independently. Following an occurrence of target touching, the experimenter delivered the consequence associated with the selected response option (HP social interaction, LP social interaction, or no interaction) for 3–5 s. Exposure trials continued until the participant independently emitted the target-touching response for five consecutive trials.

During sessions, participants were seated at a table with two or three response targets available, depending on the phase. For each response option, the response targets were two colored cards (12.7 × 17.8 cm) that were affixed to a board. Each target was assigned a distinct color that was consistently associated with a specific consequence across phases and across the three generality assessments. For example, one color was associated with the HP social interaction, another with the LP social interaction, and another with the control condition.

The experimenter quasi-randomly alternated the spatial position of the targets (left, center, right) across sessions. During reinforcement phases only, cards depicting the HP social interaction, LP social interaction, or control were presented above the corresponding targets to ensure differential stimulus-consequence associations.

### 4.1. Baseline

During three-response-option baseline phases, three targets associated with HP, LP, and control consequences were available without the corresponding picture cards, and no programmed consequences were delivered for target touching. During two-response-option baseline phases, only the LP and control targets were available without the corresponding picture cards, and no programmed consequences were delivered for target touching. The experimenter maintained a neutral expression, and the targets remained on the table in front of the participant throughout baseline sessions. The purpose of the two-response-option baseline was to assess baseline responding for the LP option when the HP alternative was not concurrently available and to provide a comparison condition for the subsequent two-response-option reinforcement phase.

### 4.2. Reinforcement

During the three-response-option phases, target touching produced differential consequences depending on the selected option. The picture cards that corresponded with each response option were presented above the targets. Target touching on the HP or LP target resulted in delivery of the corresponding social interaction for 3–5 s on a fixed-ratio (FR) 1 schedule. Target touching on the control target resulted in no programmed consequences; the experimenter made no change in posture or facial expression and continued to look away from the participant.

During the two-response-option reinforcement phases, only the LP and control targets were available. Target touching on the LP option produced the corresponding LP social interaction for 3–5 s on an FR 1 schedule, whereas target touching on the control option resulted in no programmed consequences. The purpose of this phase was to determine the absolute reinforcing efficacy of the LP social interaction.

### 4.3. Results and Discussion

**Figure 2:**
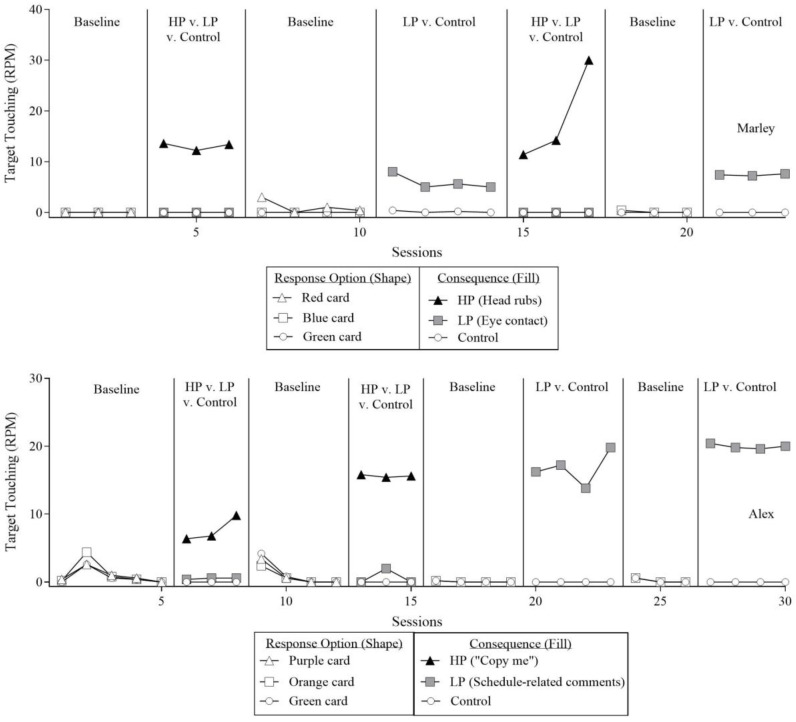
The results of Reinforcer Assessment 1: Simple Arbitrary Task, comparing rate of target touching. Note. HP = highly preferred; LP = less preferred.

Figure 2 depicts the results of Reinforcer Assessment 1: Simple Arbitrary Task for both participants. The shapes in the figure correspond to the three different response options, and the fill of the shapes indicates the consequences available during each phase. The white, black, and gray shapes indicate no programmed consequences, HP social interaction, and LP social interaction, respectively. Marley (top panel) did not exhibit target touching during the initial three-response-option baseline (*M* = 0 rpm). During the subsequent three-response-option reinforcement (HP, LP, control) phase, when the HP social interaction, the LP social interaction, and control were concurrently available, Marley exhibited high levels of target touching (*M* = 13.1 rpm) for the HP social interaction (head rubs) and zero levels of target touching for the LP social interaction (eye contact) and control options (*M*s = 0 and 0 rpm, respectively). During the return to the three-response-option baseline phase, target touching decreased to low levels across HP, LP, and control options (*M*s = 1.1, 0, and 0 rpm, respectively). When the two-response-option reinforcement phase (LP, control) was implemented, Marley exhibited high levels of target touching for the LP social interaction (*M* = 5.9 rpm) and low levels for the control option (*M* = 0.2 rpm). When the three-response-option reinforcement phase was subsequently reimplemented, the initial pattern of results was replicated. Marley exhibited high levels of target touching for the HP social interaction (*M* = 18.5 rpm) and zero levels for the LP or control options (*M*s = 0 and 0 rpm, respectively). During the return to the two-response-option baseline phase, low levels of target touching were observed across LP and control options (*M*s = 0.1 and 0 rpm, respectively). During the subsequent two-response-option reinforcement phase, the initial findings were replicated, with high levels of target touching for the LP social interaction (*M* = 7.4 rpm) and zero levels for the control option (*M* = 0 rpm).

Alex (bottom panel) exhibited low levels of target touching during the initial three-response-option baseline phase (*M*s = 1, 1.2, and 0.7 rpm for HP, LP, and control options, respectively). During the subsequent three-response-option reinforcement phase (HP, LP, control), Alex exhibited high levels of target touching for the HP social interaction (*M* = 7.7 rpm) and low levels of responding for the LP and control options (*M*s = 0.5 and 0 rpm, respectively). These results were replicated in the subsequent three-response-option baseline and reinforcement phases, with low levels of target touching across response options during baseline (*M*s = 1, 0.8, and 1.3 rpm respectively) and higher levels for the HP option during reinforcement (*M* = 15.6 rpm) relative to the LP and control options (*M*s = 0.7 and 0 rpm, respectively).

Following these phases, four two-response option phases were conducted. During the initial two-response-option baseline phase, low levels of target touching were observed for both the LP and control options (*M*s = 0.1 and 0.1 rpm, respectively). During the subsequent two-response-option reinforcement phase, Alex exhibited high levels of target touching for the LP option (*M* = 16.8 rpm) and zero levels for the control option (*M* = 0 rpm). These results were replicated during the subsequent two-response-option baseline and reinforcement phases, with low levels of responding during baseline (*M*s = 0.2 and 0.2 rpm for LP and control, respectively) and high levels of responding for the LP option during reinforcement (*M* = 20 rpm), with zero levels for the control option (*M* = 0 rpm).

For both participants, HP and LP social interactions functioned as reinforcers for target touching. Although the HP social interaction resulted in higher levels of target touching relative to the LP social interaction during the three-response-option reinforcement phases, the LP social interaction functioned as a reinforcer during the two-response-option reinforcement phases. Thus, the presence of the HP social interaction masked the reinforcing efficacy of the LP social interaction when both options were concurrently available. Without the inclusion of the two-response-option reinforcement phases, the reinforcing efficacy of the LP social interaction may have been overlooked, resulting in a false negative outcome.

For Alex, the LP social interaction produced higher rates of target touching in the two-response-option reinforcement phase than those observed for the HP social interaction during the three-response-option reinforcement phase. One possible explanation is that Alex allocated responding across multiple options when both HP and LP social interactions were concurrently available. As a result, these findings should be interpreted with caution, as a two-response-option reinforcement phase comparing HP and control was not conducted. Additionally, target touching was a relatively low-effort response that was already in the participants’ repertoires prior to the study. Therefore, it remains unclear whether similar outcomes would be obtained for responses requiring greater effort or complexity. To address this limitation, two subsequent reinforcer assessments were conducted to evaluate the generality of the reinforcing effects of HP and LP social interactions for more complex skills involving multistep response chains.

## 5. Reinforcer Assessment 2: Complex Arbitrary Task

### 5.1. Procedures

The purpose of this reinforcer assessment was to evaluate the reinforcing efficacy of the HP and LP social interactions for completing complex arbitrary tasks (i.e., eight-step response chains to construct LEGO structures). An adapted alternating treatments design was used to compare concurrent interventions across functionally independent skills of equal response difficulty (Holcombe et al., 1994; Sindelar et al., 1985). This design allowed evaluation of the relative reinforcing effects of HP, LP, and control consequences on task acquisition. As an additional control, the assessment was conducted twice with each participant to evaluate the reliability of outcomes and to help account for differences in task complexity. A multiple baseline across tasks with an embedded multielement design was used to evaluate the rate of learning across the HP, LP, and control LEGO structures. Three distinct LEGO structures were taught to mastery before a second group of three distinct LEGO structures were introduced to participants to see the replication of the outcomes within participants.

During each assessment, three novel LEGO structures were used as target tasks. Each structure consisted of an eight-step response chain, with each step requiring the participant to select a specific LEGO piece and place it in the correct location relative to other pieces (See examples in Figure S1 in Supplementary Materials). The experimenter constructed the LEGO structures and attempted to equate response difficulty across steps and across structures. Each structure was randomly assigned to the HP, LP, or control condition using a random number generator.

The baseplates and LEGO pieces for each structure were color-coded to correspond with the HP, LP, and control response options used in Reinforcer Assessment 1. For example, if the HP response option was associated with a red target in Reinforcer Assessment 1, the baseplate and LEGO pieces associated with the HP social interaction were red. During each trial, the experimenter presented one LEGO structure. Trials were alternated in a quasi-random sequence, such that each structure was presented once before any structure was repeated (e.g., HP, control, LP, control, LP, HP). Sessions lasted approximately 60 min, and were conducted 2–3 times per week, consisting of 10–20 trials per session.

The mastery criterion for each LEGO structure was independent completion of the eight-step response chain across two consecutive trials. Once mastery was achieved for one structure, the experimenter initiated a subsequent phase in which the most effective consequence identified for the mastered task (HP, LP, or control) was delivered for the remaining two unmastered structures. This phase assessed whether the introduction of the most effective consequence would result in mastery of the remaining tasks.

Observers used paper-and-pencil recording to score the occurrence or nonoccurrence of correct unprompted responses for each step of the task analysis. A correct unprompted response was defined as completion of a step in the appropriate sequence prior to the delivery of a prompt during a trial. A nonoccurrence was defined as completion of a step following a prompt, completion of a step out of sequence, or the omission of a step. Data were summarized as the total number of correct unprompted steps completed per trial.

Interobserver agreement (IOA) was assessed by a second observer independently scoring each trial. Agreement was defined as both observers scoring the same outcome (occurrence or nonoccurrence) for each step with a response opportunity. For example, when training was in effect for Step 3, observer records were compared for Steps 1, 2, and 3. IOA was calculated by dividing the total number of agreements by the total number of agreements and disagreements and multiplying by 100. IOA data were collected for 41.5% of trials for Marley and 41.4% of trials for Alex. Mean IOA was 98.5% (range: 75–100%) for Marley and 99.6% (range: 80–100%) for Alex.

### 5.2. Baseline

During baseline trials, the experimenter presented the LEGO baseplate and pieces for one structure and delivered the instruction, “Let’s build LEGOs.” No prompts were delivered following the instruction, and the corresponding consequence cards were not presented. No programmed consequences were delivered for correct or incorrect responding. Trials ended when the participant placed all pieces on the baseplate or did not respond for 30 s.

### 5.3. Training

Training procedures were identical to baseline with the addition of prompting and reinforcement components. At the start of each trial, the experimenter presented the LEGO materials for one structure and delivered the instruction, “Let’s build LEGOs.” Cards depicting the HP social interaction, LP social interaction, or control condition were placed next to the corresponding LEGO materials to establish differential stimulus–consequence relations.

The steps were taught using forward chaining without the completion of untrained steps. Training began with the first step not completed independently during baseline (the first step for all structures and both participants). Each training step was taught until the participant demonstrated an independent correct response across two consecutive trials, after which training progressed to the next step in the chain. During trials, the experimenter blocked attempts to complete untrained steps, allowing participants to complete only previously mastered steps and the current training step. As a result, the number of opportunities per trial ranged from one to eight, depending on the step currently targeted for training. A 30 s intertrial interval followed each trial.

The prompting component was implemented during the training phase across all LEGO structures (HP, LP, and control). During the prompting component, the experimenter used a progressive time-delay procedure consisting of an immediate prompt, a 2 s delayed prompt, and no prompt, consistent with the most effective procedure identified by Seaver and Bourret (2014). The prompt hierarchy started with an immediate prompt on the initial training trial, and the prompt was faded to 2 s delay following two consecutive trials with a correct prompted response. The prompt was faded to no prompt following two consecutive trials with a correct response, prompted or independent. The experimenter increased the intrusiveness of prompting (i.e., no prompt to 2 s delay prompt, or 2 s delay prompt to an immediate prompt) following two consecutive trials with completion of a step out of sequence or the omission of a step. During immediate-prompt trials, the experimenter physically guided completion of the training step immediately following the instruction. During the 2 s delay trials, the experimenter gestured toward the correct LEGO piece if the participant did not respond independently within 2 s; physical guidance was provided if the participant did not respond following the gesture. During no-prompt trials, if the participant did not complete the step within 10 s, the experimenter implemented a correction procedure consisting of physical guidance. During the reinforcement component, the experimenter delivered the consequence associated with the LEGO structure (HP social interaction, LP social interaction, or no interaction) for 3–5 s following prompted or independent completion of the training step.

### 5.4. Results and Discussion

**Figure 3:**
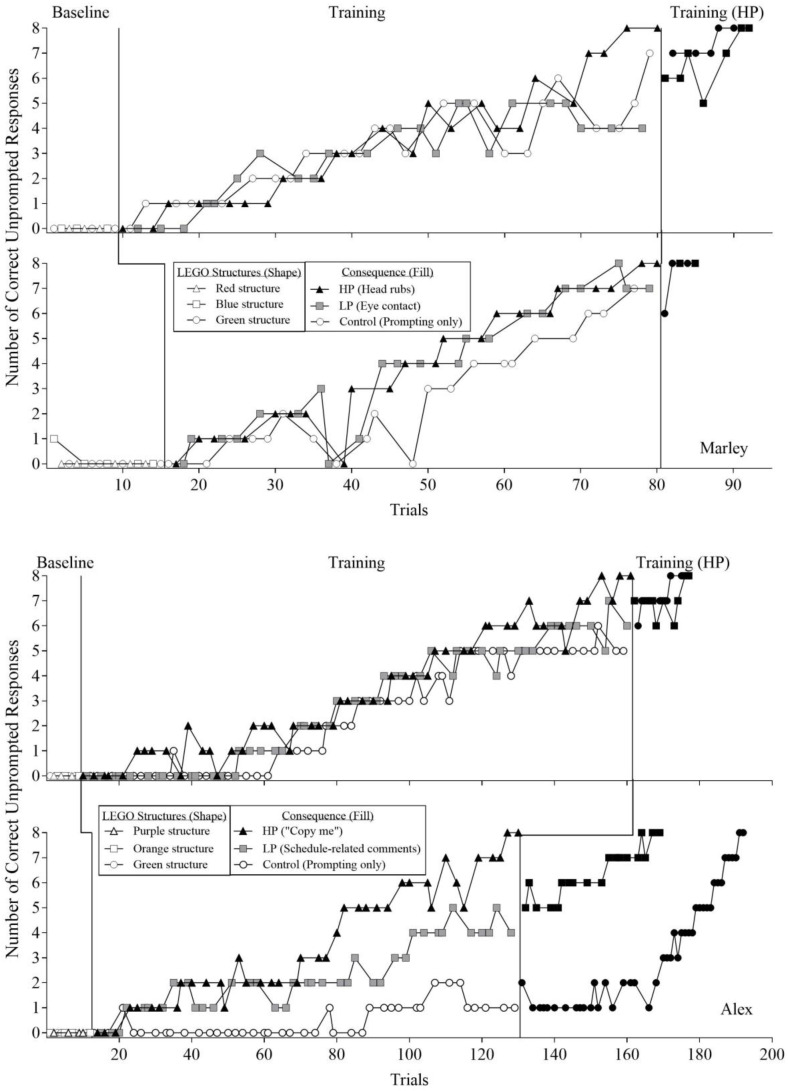
The results of Reinforcer Assessment 2: Complex Arbitrary Task. Note. HP = highly preferred; LP = less preferred.

Figure 3 shows the noncumulative trial-by-trial data across baseline and training phases. The total number of correct unprompted responses during each trial for Marley and Alex is depicted in Figure 3. The shapes in the figure correspond to the three different LEGO structures, and the fill of the shapes indicates the consequences available during each phase. The white, black, and gray shapes indicate no programmed consequences, HP social interaction, and LP social interaction, respectively. In addition, Table 2 shows the total number of trials for the LEGO structure response chains to reach mastery criterion (i.e., two consecutive trials with correct and independent completion of all eight steps). Both participants met the mastery criterion with the HP structure prior to meeting the mastery criterion for the LP and control structures.

For Marley, responding was undifferentiated across conditions during the first assessment (Figure 3, top panel) until approximately Trial 70, at which point responding for the HP structure became clearly differentiated. Marley met the mastery criterion for the HP structure at Trial 80. When the HP social interaction was subsequently delivered contingent on responding for the LP and control structures, both structures reached mastery achieved within 12 and 10 trials, respectively. In the second assessment (Figure 3, second panel), responding was initially similar across structures. Differential responding emerged around Trial 40, with higher levels of correct unprompted responding for the HP and LP structures relative to the control structure. Marley met mastery for the HP structure at Trial 80, whereas the LP and control structures remained at seven correct steps. When the HP consequence was subsequently delivered for correct responding on the LP and control structures, both structures’ mastery was achieved within five and four trials, respectively.

For Alex’s first assessment (Figure 3, third panel), correct unprompted responding for the HP and LP structures exceeded that observed for the control structure until approximately Trial 120, at which point responding for the HP structure increased above both the LP and control structures, which remained at five to six correct unprompted steps. Alex met mastery for the HP structure at Trial 161. When the HP social interaction was subsequently delivered contingent on correct responding for the LP and control structures, both structures were acquired within 15 and 14 trials, respectively. In the second assessment (Figure 3, bottom panel), an increasing trend in correct unprompted responding was observed for the HP and LP structures relative to the control structure. Differential responding emerged around Trial 70, with higher levels of responding for the HP structure relative to the LP structure. Alex met mastery for the HP structure at Trial 130. When the HP consequence was subsequently delivered contingent on correct responding for the LP and control structures, responding on the LP structure increased, and mastery was achieved within 39 trials. Responding on the control structure remained relatively stable until approximately Trial 170, after which an increasing trend was observed, and mastery was achieved after 62 trials.

This reinforcer assessment evaluated the generality of the preference assessment outcomes using a dependent variable of increased complexity (i.e., response effort and response variability). For Marley, who exhibited higher levels of target touching for the HP option relative to the LP option during Reinforcer Assessment 1, similar outcomes were obtained during Reinforcer Assessment 2. Specifically, Marley met mastery for the HP structure prior to the LP and control structures. As with Marley, Alex’s results in Reinforcer Assessment 2 were consistent with outcomes obtained from Reinforcer Assessment 1. Alex met mastery for the HP structure before meeting mastery for the LP and control structures. These findings indicate that preference assessment outcomes predicted the relative reinforcing effects of HP and LP social interactions for a more complex arbitrary task involving a multistep response chain.

In summary, the findings from Reinforcer Assessment 2 were consistent with the relative reinforcing effects observed during Reinforcer Assessment 1 for both participants. However, an additional finding from Reinforcer Assessment 1 was that the LP social interaction functioned as an absolute reinforcer for target touching when it was the only available social consequence. The current experimental design did not permit evaluation of whether the LP social interaction would have resulted in the acquisition of the LEGO structures with extended training. Because extended exposure to the LP consequence was not conducted following mastery of the HP structure, the absolute reinforcing efficacy of the LP social interaction for this complex task remains unclear.

Although the results of Reinforcer Assessment 2 support the generality of PSPA outcomes to complex arbitrary response chains, the extent to which similar findings would be obtained for socially relevant complex response chains has not yet been determined. The LEGO tasks taught in this assessment consisted of steps with similar topographies and levels of difficulty (i.e., picking up and placing LEGO pieces). In contrast, socially relevant skills often involve response chains with greater variability in step difficulty and topography. Thus, the generality of PSPA outcomes to socially relevant complex response chains remains to be determined.

## 6. Reinforcer Assessment 3: Complex Socially Relevant Task

### 6.1. Procedures

This reinforcer assessment was identical to Reinforcer Assessment 2: Complex Arbitrary Task, with the exception that the response chains were socially relevant skills identified by caregivers. To identify socially relevant tasks, the experimenter interviewed two caregivers using a questionnaire that listed vocational and hygiene tasks. For each task, the experimenter asked the caregiver to indicate whether the participant could complete the task independently (I), with prompting (P), or could not complete the task with prompting (C). For tasks scored as P or C, caregivers were asked to indicate whether the skill was important for the participant to learn (yes or no). Caregivers were asked to list additional socially relevant tasks in an open-ended portion of the questionnaire, and caregivers again reported whether the participant could complete the task independently, with prompting, or could not complete the task with prompting. For Alex, several tasks related to using an iPad were identified through this open-ended portion. Caregivers were then asked to rank order all tasks scored as both P and yes from most to least beneficial. 

Based on the results of this questionnaire, six socially relevant tasks were identified for each participant (see Table 3). For Marley, these tasks included cleaning a room, alphabetizing folders, filing notecards, stuffing envelopes, collating and stapling paper, and rolling silverware. For Alex, these tasks included collating and stapling paper, folding a fitted sheet, making a first aid kit, editing and cropping photos, adding an event to a calendar, and adding a phone number to a contact list.

A multiple baseline across tasks with an embedded multielement design (i.e., trials were alternated in quasi-random order) was used to evaluate the rate of learning across the HP, LP, and control tasks. Three tasks were taught to mastery before the next three tasks were introduced to participants to assess the replication of the outcomes within participants. The experimenter developed a task analysis for each task by dividing the skill into eight steps (see Tables S1 and S2 in Supplementary Materials). For each participant, the reinforcer assessment was conducted twice to evaluate the reliability of outcomes. Each assessment included three of the six identified tasks. Tasks were grouped and assigned to the first or second assessment based on overall difficulty and response modality. For example, a vocal response was included in each of the three task analyses of the first assessment with Marley. The prompting procedures during the training phase were identical to those used in Reinforcer Assessment 2.

Response measurement and data collection procedures were identical to those used in Reinforcer Assessment 2. Observers used paper-and-pencil recording to score the occurrence or nonoccurrence of correct unprompted responding for each step of the task analysis. A correct unprompted response was defined as completion of a step in the appropriate sequence prior to the delivery of a prompt during a trial. A nonoccurrence was scored if the participant completed a step following a prompt, completed a step out of sequence (e.g., completing Step 3 before Step 2), or omitted a step. Data were summarized as the total number of correct unprompted steps completed per trial. Sessions lasted approximately 60 min and were conducted 2–3 times per week, consisting of 10–20 trials per session.

A second observer independently collected trial-by-trial data. Trial-by-trial IOA was collected for 33.9% of trials for Marley and 38.3% of trials for Alex. The mean agreement for steps scored as occurrences and nonoccurrences of correct unprompted responding was 94.9% (range: 50–100%) for Marley and 99.7% (range: 83.3–100%) for Alex.

### 6.2. Results and Discussion

**Figure 4:**
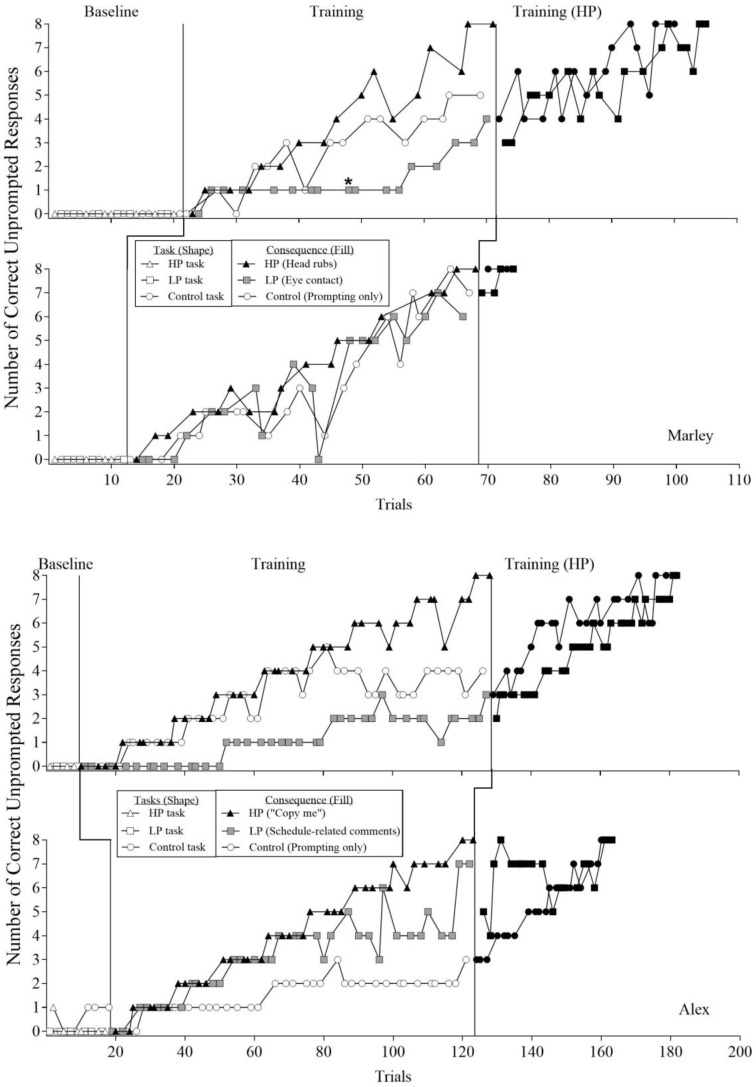
The results of Reinforcer Assessment 3: Complex Socially Relevant Task Note. Asterisk indicates when prompt type was modified at Step 2 for the LP task for Marley.

Figure 4 shows the noncumulative trial-by-trial data across baseline and training phases. Figure 4 depicts the total number of correct unprompted steps completed during each trial for Marley (top two panels) and Alex (bottom two panels), respectively. The shapes in the figure correspond to the three different socially relevant tasks, and the fill of the shapes indicates the consequences available during each phase. The white, black, and gray shapes indicate no programmed consequences, HP social interaction, and LP social interaction, respectively. For both participants, the task associated with the highly preferred (HP) social interaction resulted in the most efficient acquisition of the socially relevant response chains. Table 4 shows the total number of trials for the socially relevant tasks to reach the mastery criterion (i.e., two consecutive trials with correct and independent completion of all eight steps).

For Marley’s first assessment (Figure 4, top panel), responding to the less preferred (LP) task did not advance beyond Step 2. Because this step required a vocal response, the experimenter hypothesized that the existing model prompt (i.e., “G”) was insufficient to occasion correct responding. Consequently, the prompt was modified from a model to a clear instruction (i.e., the experimenter stated “say G”). Following this modification, a correct unprompted response for this step occurred within three trials. This modified prompt was subsequently used for the vocal response step in both the LP and control tasks. Marley’s correct unprompted responding initially increased for both the HP and control tasks. Around Trial 45, responding on the HP task increased and became differentiated from the control task. Marley met the mastery criterion for the HP task first, at Trial 71. When the HP consequence was subsequently delivered contingent on correct responding for the LP and control tasks, acquisition occurred for both tasks, with mastery achieved within 34 and 29 trials, respectively.

In Marley’s second assessment (Figure 4, second panel), an increasing trend in correct responding was observed across all three tasks. A brief decrease in responding for the LP and control tasks occurred between Trials 35 and 50, corresponding with the nonoccurrence of correct unprompted responses for previously mastered steps. Nevertheless, Marley again met mastery first for the HP task, at Trial 68. When the HP consequence was applied to the LP and control tasks, mastery was achieved within six and five trials, respectively.

For Alex’s first assessment (Figure 4, third panel), correct unprompted responding increased and was consistently higher for the HP and control tasks relative to the LP task until approximately Trial 80. During this period, responding to the control task stabilized at Step 2 until mastery of the HP task was achieved at Trial 128. When the HP social interaction was subsequently delivered for correct responding on the LP and control tasks, both tasks showed increasing trends, and mastery was achieved within 54 and 51 trials, respectively.

In Alex’s second assessment (Figure 4, bottom panel), correct unprompted responding was higher for the HP and LP tasks relative to the control task. Beginning around Trial 75, responding on the HP task exceeded that observed for the LP task and remained differentiated until the mastery criterion was met at Trial 123. When the HP consequence was then applied to the LP and control tasks, Alex met mastery for the LP and control tasks within 40 and 39 trials, respectively.

Across both participants, this reinforcer assessment extended the generality of the PSPA outcomes to socially relevant response chains characterized by greater response variability. Consistent with earlier assessments, the HP social interaction produced the most efficient acquisition of novel skills. These findings demonstrate that highly preferred forms of social interaction can function as effective reinforcers for teaching socially relevant multistep skills to individuals with ASD and suggest that relative preference for social interaction may influence rates of skill acquisition. During a 3-month follow-up interview, Alex’s caregiver reported that performance on the socially relevant skills acquired during this assessment was maintained in the natural environment. Follow-up data were not available for Marley.

Although HP social interactions generally produced more efficient acquisition, the magnitude of differentiation between HP, LP, and control tasks varied across assessments and tasks. In Marley’s first assessment and Alex’s second assessment, the mastery was achieved substantially more rapidly for HP tasks relative to LP and control tasks. In contrast, differences in acquisition rates were less pronounced in Marley’s second assessment and Alex’s first assessment. These findings suggest that the reinforcing efficacy of HP social interactions, relative to LP or control conditions, may vary as a function of task characteristics. The specific task features contributing to this variability were not isolated and warrant further investigation.

## 7. General Discussion

The purpose of the current study was to assess the generality of outcomes from a paired-stimulus preference assessment (PSPA) for identifying social interaction as a reinforcer across target skills of increasing complexity and social relevance. Across three reinforcer assessments, highly preferred (HP) social interactions consistently produced more efficient acquisition than less preferred (LP) social interactions and a no-interaction control. These findings extend previous research by demonstrating that PSPA outcomes can predict relative reinforcing effects not only for simple arbitrary responses, but also for complex arbitrary and socially relevant response chains.

Results from Reinforcer Assessment 1 replicated and extended prior findings by showing that both HP and LP social interactions functioned as reinforcers for a simple target response (Kelly et al., 2014; Lang et al., 2014; Morris & Vollmer, 2019). However, LP social interactions were less effective when HP alternatives were concurrently available, indicating that the absolute reinforcing efficacy of lower-preference social interactions may be difficult to determine in a concurrent arrangement. Similarly to previous research with pictorial preference assessments for social interaction, the relative preference of social interactions was indicative of the relative reinforcing efficacy during reinforcer assessments (e.g., Davis et al., 2022; Harper et al., 2021). These findings highlight the importance of assessing both relative and absolute reinforcing effects when evaluating social interaction as a consequence, particularly when higher-preference alternatives may not always be available in applied settings (e.g., Goldberg et al., 2023).

Reinforcer Assessment 2 demonstrated that HP social interactions produced more efficient acquisition of complex arbitrary response chains relative to LP and control conditions. For both participants, mastery was achieved first for tasks associated with HP social interactions, indicating that PSPA outcomes predicted relative reinforcing effects for response chains requiring greater response effort and variability. These findings replicate those of Morris and Vollmer (2020c) by showing that HP social interactions can facilitate faster acquisition of complex skills compared to those of lesser preferred social interactions. However, because extended exposure to LP social interactions was not evaluated following mastery of HP-associated tasks, the absolute reinforcing efficacy of LP social interactions for complex arbitrary skills remains unclear. In addition, it is unclear if the control (prompting only) tasks would have reached mastery criterion without training with HP social interaction. It is possible that the HP social interaction enhanced the efficiency of learning with the LP and control response chains. The effects of combining HP social interactions with other effective teaching components (e.g., prompt types) should be evaluated in future research.

Reinforcer Assessment 3 further extended these findings to complex socially relevant response chains and extended the findings of Morris and Vollmer (2020c) by demonstrating the generality of social interactions as reinforcers when teaching complex skills. Across participants, HP social interactions again resulted in the most efficient acquisition of novel socially relevant skills. Although LP social interactions supported acquisition when HP interactions were delivered following mastery, acquisition was generally slower and less consistent relative to HP interactions. It is noteworthy that the outcomes of Reinforcer Assessment 2 aligned with those of Reinforcer Assessment 3, showing the fastest acquisition with the HP social interactions. Similarly to Seaver and Bourret (2014), the most effective intervention components for teaching the arbitrary tasks were also most effective for teaching the socially relevant tasks. These findings suggest that relative preference for social interaction may influence rates of skill acquisition for socially relevant tasks and underscore the value of systematically identifying highly preferred forms of social interaction prior to instruction.

There were a couple of limitations of this study that should be considered in future areas of research. First, both participants in this study communicated vocally and demonstrated conditional discrimination skills during an auditory-to-visual matching-to-sample task. Perhaps these skills could have influenced the rate of learning complex skills. By contrast, Morris and Vollmer (2020c) included participants who communicated using augmentative and alternative communication (AAC) and used the SIPA to identify the consequences for the reinforcer assessment. Although there were differences in the communication modalities across participants in Morris and Vollmer and the present study, the HP social interaction resulted in the fastest rates of skill acquisition in both studies. It may be beneficial to develop an efficient pre-assessment of individual skills to determine the optimal preference assessment format for identifying social interactions in future research. This may be particularly important for individuals for whom the HP social interaction differs across preference assessment formats (e.g., Morris & Vollmer, 2020a) because the relative reinforcing efficacy could impact the rate of skill acquisition. Second, there were fewer trials to mastery for some of the control response chains relative to the LP response chains in Reinforcer Assessment 2 and Reinforcer Assessment 3 (see Table 2 and Table 4). There may have been carryover effects between the response chains due to the rapid alternation of trials in the multielement design. Although different colors were associated with different consequences and each response chain included a distinct set of responses, the participants experienced frequent social interaction throughout sessions. The schedule of reinforcement within a session may have been sufficient to maintain correct responding during trials with the control response chains. This could be addressed in future research by presenting trials in blocks of 10 trials with slower alternation of HP, LP, and control response chains.

Taken together, the current findings contribute to a growing literature supporting the use of social interaction as an effective reinforcer in skill acquisition programs for individuals with autism spectrum disorder. Social interaction is naturally embedded within instructional contexts, readily available, and may promote generalization and maintenance of skills across settings. Although the present results suggest that PSPAs may be useful for identifying forms of social interaction that are likely to function as effective reinforcers across a range of instructional tasks, more research on social reinforcers is needed to guide clinical practice. In a review by Morris et al. (2023), only 20 studies since 2010 were identified that included a preference assessment of social interactions. The systematic identification of preferred social interaction may lead to more efficient learning outcomes, and therefore, it appears important for clinicians to take the time to conduct systematic assessments for identifying preferred social interactions. There were several pre-assessments used in the current study, and there was overlap in the forms of social interaction identified across pre-assessments. We recommend that clinicians consider using at least one pre-assessment to inform preference assessment arrays for social interaction.

Although less preferred (LP) social interactions functioned as reinforcers under some conditions, the experimental design did not permit a systematic evaluation of whether LP social interactions would have supported the acquisition of complex skills with extended exposure. The LP and control tasks in Reinforcer Assessments 2 and 3 showed an increasing trend in skill acquisition rates with the LP social interaction and prompting only, respectively. However, the introduction of the HP social interaction for the LP and control tasks limits the conclusions regarding the absolute reinforcing efficacy of the LP social interaction for multi-step tasks. Future research should examine the effects of prolonged training with LP social interactions and evaluate conditions under which lower-preference social interactions may be sufficient to support skill acquisition. Additionally, future studies should examine task characteristics (e.g., response effort, response modality, and variability across steps) that may moderate the relative reinforcing efficacy of social interactions.

In summary, the current study demonstrates that PSPA outcomes can predict the relative reinforcing effects of social interaction across tasks of increasing complexity and social relevance. These findings highlight the importance of evaluating social interaction as a reinforcer beyond simple response classes and suggest that systematic identification of preferred forms of social interaction may enhance the efficiency of instructional programming for individuals with ASD.

## Figures and Tables

**Table 1:** Descriptions of Social Interactions Included in the PSPA Stimulus Array, by Participant.

Participant	Social Interaction (Preassessment Source)	Definition
Marley	Arm tickles (IA)	Experimenter touches the participant’s forearms with alternating fingertips of both hands using a light touch, moving from elbow to wrist and vice versa.
	Head rubs (IA)	Experimenter moves fingertips along the participant’s scalp with a light touch from front to back and vice versa.
	Preferred non-work topics (IA/MMO)	Experimenter makes a comment or asks a question related to subject matter other than a work schedule or contingency, 1–2 conversational exchanges. Topics were identified by the participant prior to sessions.
	Praise (DA)	Experimenter delivers a verbal statement of affirmation (e.g., “That’s cool!” or “Good job!”) paired with a thumbs-up hand gesture.
	Schedule-related topics (DA/MMO)	Experimenter comments on or asks a question related to past or upcoming events for 1–2 conversational exchanges.
	Eye contact (DA)	At a distance of 1 m or less, the experimenter orients toward the participant and looks into the participant’s face or eyes.
Alex	“Copy me” (IA/DA)	Experimenter repeats a phrase spoken by the participant while imitating the same tone and inflection. This interaction may or may not follow a participant request to “copy me.”
	Silly sounds (DA)	Experimenter names an object and produces a corresponding sound (e.g., “hair dryer—woosh”).
	Tickles (IA)	Experimenter moves fingers along the participant’s ribs and armpits.
	High-fives (IA)	Experimenter raises a hand with palm facing the participant; if the participant raises their hand, the experimenter makes contact between palms.
	Praise (DA)	Experimenter delivers a verbal statement of affirmation (e.g., “Nice work” or “You did a great job at X”) paired with a thumbs-up gesture.
	Eye contact (MMO)	At a distance of 1 m or less, the experimenter opens eyes wide and looks directly into the participant’s eyes.
	Schedule-related comments (MMO)	Experimenter makes a statement or responds to a question related to planned current or future events (e.g., “first X, then Y”).

Note. IA = indirect assessment; DA = descriptive assessment; MMO = manipulation of motivating operation.

**Table 2:** The total number of trials to achieve the mastery criterion for task analyses in Reinforcer Assessment 2.

		Trials to Mastery		
Participant	Task Analysis	HP	LP	Control
Marley	LEGO Structure 1	27	33 (7)	32 (5)
	LEGO Structure 2	27	29 (2)	29 (3)
Alex	LEGO Structure 1	25	32 (9)	31 (7)
	LEGO Structure 2	44	61 (19)	87 (43)

Note. The number in parentheses indicates the number of trials to mastery that included the HP social interaction during training.

**Table 3:** Tasks used in Reinforcer Assessment 3: Complex Socially Relevant Task.

Participant	Set 1 Tasks	Set 2 Tasks
Marley	HP—Cleaning a room	HP—Envelope stuffing
	LP—Alphabetizing folders	LP—Collating and stapling paper
	Control—Notecard filing	Control—Rolling silverware
Alex	HP—Collating and stapling paper	HP—Edit and crop photo
	LP—Folding a fitted sheet	LP—Add event to calendar
	Control—Making first aid kit	Control—Adding phone number to contact list

**Table 4:** The total number of trials to achieve the mastery criterion for task analyses in Reinforcer Assessment 3.

		Trials to Mastery		
Participant	Task Analysis	HP	LP	Control
Marley	Task Set 1	24	43 (19)	38 (15)
	Task Set 2	23	26 (4)	25 (2)
Alex	Task Set 1	43	73 (30)	66 (24)
	Task Set 2	41	60 (19)	62 (21)

Note. The number in parentheses indicates the number of trials to mastery that included the HP social interaction during training.

## Data Availability

The data presented in this study are available on request from the corresponding author due to privacy restrictions.

## Supplementary Materials

The following supporting information can be downloaded at: https://www.mdpi.com/article/10.3390/bs16040543/s1, Figure S1: Example of Completed Lego Structures during Reinforcer Assessment 2; Table S1: Task Analyses from Reinforcer Assessment 3 for Marley; Table S2: Task Analyses from Reinforcer Assessment 3 for Alex.
