# Supplementary material for: Generality of a Paired-Stimulus Preference Assessment for Identifying Reinforcing Forms of Social Interaction
Source: Behav Sci (Basel). 2026 Apr 6;16(4):543. doi: 10.3390/bs16040543 (PMC13113614; doi:10.3390/bs16040543)
Supplement: Supplementary file 1 [file behavsci-16-00543-s001.zip › behavsci-4101101-supplementary.pdf]

Supplemental Information

Table S1
Task Analyses from Reinforcer Assessment 3: Complex Socially Relevant Task for Marley

Set 1 Tasks

Cleaning a room (HP)
1. Pick up crumpled paper
2. Place paper in trash can
3. Place paper clips into container
4. Place lid on container
5. Pick up one white plastic folder
6. Walk to location within 1 m of second folder
7. Vocally state aloud the number on folder
8. Place folder inside second folder

Alphabetizing folders (LP)
1. Touch white box with letter
2. Vocally state aloud the letter
3. Pick up dry erase marker
4. Circle the corresponding letter in the alphabet grid
5. Underline the letter to the left of circled letter
6. Touch the underlined letter
7. Walk to shelf
8. Place folder onto shelf to the right of the folder with the underlined letter

Filing notecards (Control)
1. Pick up card and walk to location with the file box
2. Open file box
3. Touch the *month* on card
4. Open to corresponding month on the dividing label in file box
5. Touch the letter marked on the card
6. Vocally state aloud the letter
7. Open to corresponding letter in file box
8. Place card in front of divider

Set 2 Tasks

Stuffing envelope (HP)
1. Place sheet of paper on top of trifold guide
2. Place lower flap of trifold guide over paper
3. Fold right side of paper and smooth edge
4. Fold left side of paper and smooth edge
5. Remove paper from guide
6. Place folded paper into envelope
7. Affix sticker to seal envelope flap
8. Add envelope to *completed* bin

Collating and stapling (LP)
1. Place one folder in work area
2. Pick up one page of document 1 and place on top of folder
3. Pick up one page of document 2 and place on top of document 2
4. Pick up one page of document 3 and place on top of document 3
5. Pick up all 3 sheets and tap on table to straighten
6. Staple top left corner of all 3 sheets
7. Place stapled sheets into folder
8. Place folder onto stack of completed folder

Rolling silverware (Control)
1. Retrieve one Ziplock from box
2. Pick up one fork and place into bag
3. Pick up one spoon and place into bag
4. Pick up one knife and place into bag
5. Pick up one napkin and place into bag
6. Zip bag closed
7. Retrieve one label and place on center of bag
8. Add napkin to *completed* bin

Table S2
Task Analyses from Reinforcer Assessment 3: Complex Socially Relevant Task for Alex

Set 1 Tasks

Set 2 Tasks

Collating and stapling (HP)

1. Place one folder in work area
2. Pick up one page of document 1 and place on top of folder
3. Pick up one page of document 2 and place on top of document 2
4. Pick up one page of document 3 and place on top of document 3
5. Pick up all 3 sheets and tap on table to straighten
6. Staple top left corner of all 3 sheets
7. Place stapled sheets into folder
8. Place folder onto stack of completed folders

Edit and crop selfie photo (HP)

1. Open camera application in iPad
2. Take selfie photo
3. Open photo
4. Click *edit*
5. Touch magic wand icon
6. Click *crop* icon
7. Crop photo of face
8. Click *done*

Folding a fitted sheet (LP)

1. Place hands in top two corners of fitted sheet
2. Put one corner over the other corner
3. Lay sheet flat on table
4. Fold bottom up to middle
5. Fold top down
6. Fold sheet in half
7. Bring sheet to closet and open door
8. Place sheet on shelf in closet

Add event to calendar (LP)

1. Open calendar application in iPad
2. Click + icon in the corner of screen
3. Type *Birthday* under event title
4. Click *all day*
5. Click *starts* and scroll to enter in corresponding date
6. Click *repeat*
7. Click *every year*
8. Click *add*

Making first aid kits (Control)

1. Retrieve one plastic bag
2. Open bag
3. Pick up one bandage and place into bag
4. Pick up one ointment and place into bag
5. Pick up one gauze pad and place in bag
6. Zip bag closed
7. Retrieve one label and place on bag
8. Place bag into *completed* bin

Add phone number to contact list (Control)

1. Open contact list on iPad
2. Click + icon in the corner of the screen
3. Click *first name* and type name corresponding to written card
4. Click *last name* and type name corresponding to written card
5. Click *company* and type company corresponding to written card
6. Scroll and click *add phone*
7. Type in phone number corresponding to numbers on written card
8. Click *done*

Figure S1

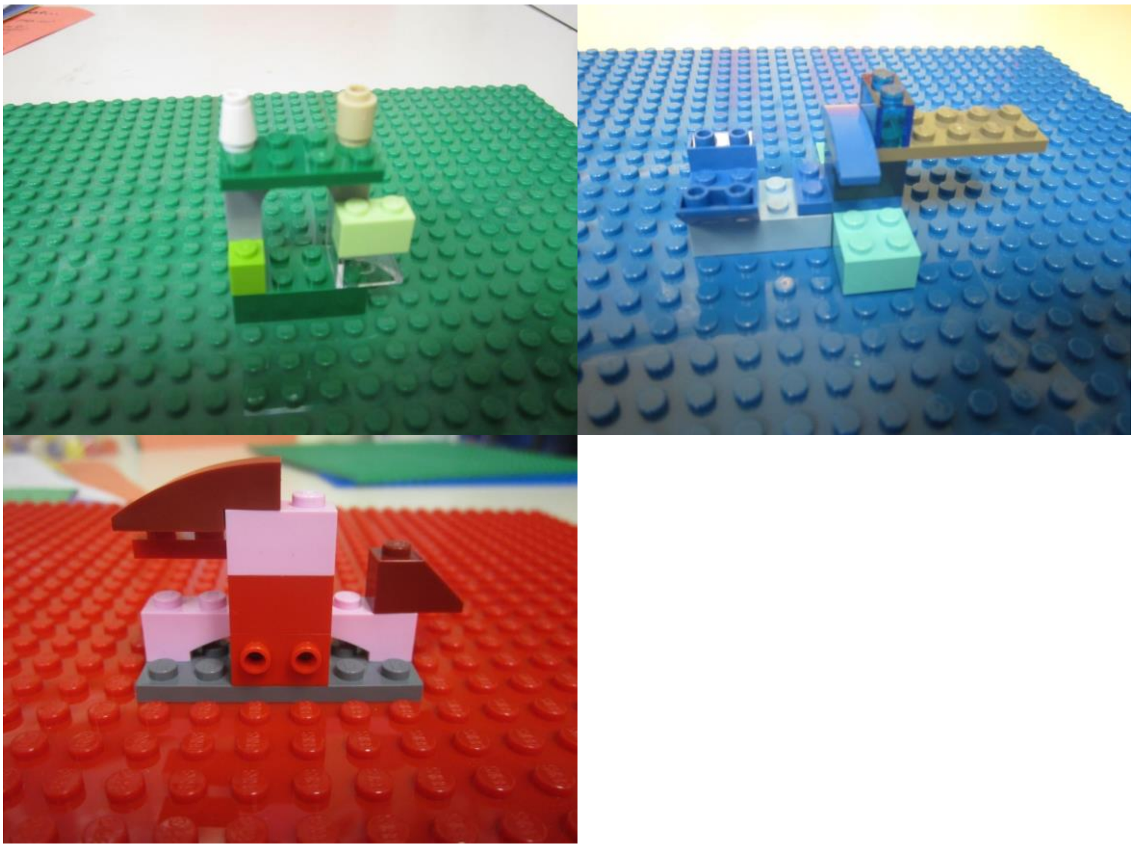

Note. This is an example of one set of completed LEGO structures used during an assessment in Reinforcer Assessment 2: Complex Arbitrary.
